# 403. Demographics, Characteristics and Health Outcomes of US Individuals Diagnosed With COVID-19 in the Outpatient Setting

**DOI:** 10.1093/ofid/ofad500.473

**Published:** 2023-11-27

**Authors:** Rikisha Gupta, Valentina Shvachko, Amos Lichtman, Mark Berry, Florentin Scarlat, Anand Chokkalingam

**Affiliations:** Gilead Sciences, Inc., Foster City, California; Gilead Sciences, Inc., Foster City, California; Gilead Sciences, Inc., Foster City, California; Gilead Sciences, Inc., Foster City, California; Gilead Sciences, Inc., Foster City, California; Gilead, Foster City, California

## Abstract

**Background:**

As the pandemic has evolved, those with COVID-19 have exhibited less severe disease, with decreased overall mortality and an increase in the proportion of individuals diagnosed in the community. However, there is a lack of data on the clinical characteristics and outcomes for nonhospitalized individuals. This descriptive analysis summarizes the demographics, clinical characteristics, and outcomes of high-risk patients diagnosed with COVID-19 in an outpatient (OP) setting.

**Methods:**

This retrospective cohort analysis used the HealthVerity database, which includes medical and pharmacy claims (commercial, Medicare, and Medicaid plans) from >330 million patients within the United States. Participants were aged ≥18 years and diagnosed with COVID-19 as OPs (study period: March 1, 2020 to November 30, 2022). Patients were followed from the index date (date of COVID-19 diagnosis) to the earliest of the following: 3 months post-index, in-hospital death, end of study period, or end of follow-up). Patients were characterized by the presence of risk factors for disease progression (RFs) at baseline and the reporting of specific COVID-19 related symptoms. COVID-19-related hospitalization and all-cause hospitalization were the evaluated outcomes.

**Results:**

In total, 15,575,934 patients met the study criteria. The median age at the index date was 50 years (IQR, 34-64), 60.6% patients were female, and 23.2% were immunocompromised (**Table 1**). Overall, 13.0% of patients had >1 COVID-19 symptom in the 7 days prior to the index date. The most commonly reported symptoms were cough (1,331,523 [8.5%]), dyspnea (1,157,567 [7.4%] and fatigue (1,063,841 [6.8%]). Most (63.1%) patients had ≥1 RF (**Figure 1**). About 11% and 14% of the patients were hospitalized within 5 and 28 days of the index date, respectively. Risk per 1000 patients for COVID-19-related hospitalization and all-cause hospitalization increased in patients as the number of RFs increased (**Table 2**).

**Figure:**
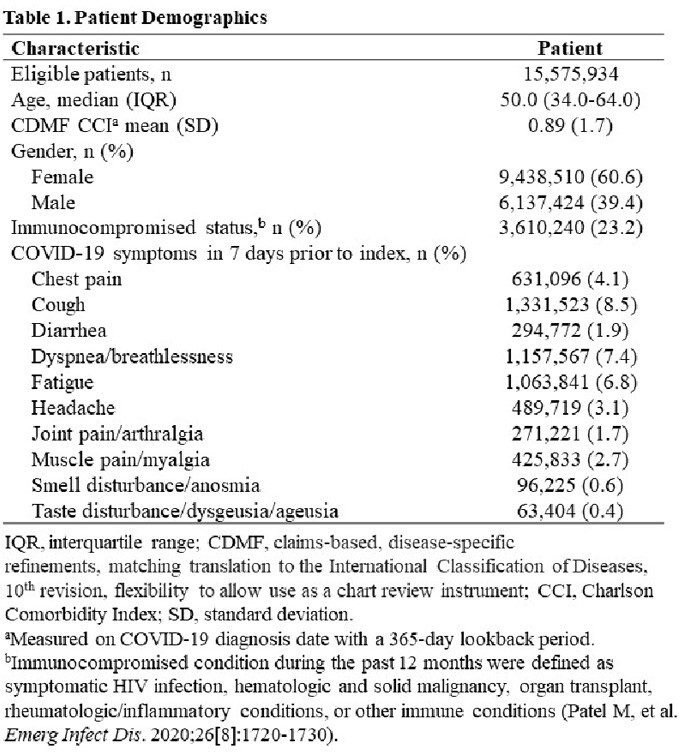

IQR, interquartile range; CDMF, claims-based, disease-specific refinements, matching translation to the International Classification of Diseases, 10th revision, flexibility to allow use as a chart review instrument; CCI, Charlson Comorbidity Index; SD, standard deviation. aMeasured on COVID-19 diagnosis date with a 365-day lookback period. bImmunocompromised condition during the past 12 months were defined as symptomatic HIV infection, hematologic and solid malignancy, organ transplant, rheumatologic/inflammatory conditions, or other immune conditions (Patel M, et al. Emerg Infect Dis. 2020;26[8]:1720-1730).

**Figure 1.**
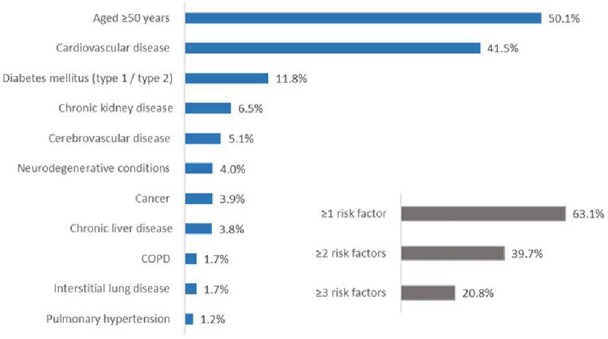
Prevalence of Risk Factors for COVID-19 Progression in Those Diagnosed with COVID-19 in an Outpatient Setting COPD, chronic obstructive pulmonary disease.

**Table 2.**
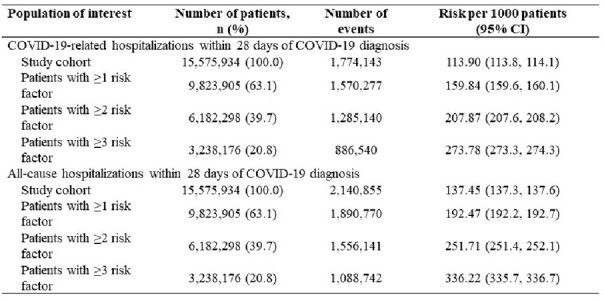
COVID-19-related Hospitalization and All-cause Hospitalization Within 28 Days of Index Date in Patients Diagnosed with COVID-19 in an Outpatient Setting, Stratified by the Number of Risk Factors

**Conclusion:**

Most patients diagnosed with COVID-19 in an OP setting had ≥1 RF; an increase in the presence of RFs was associated with an increased risk of hospitalization. The availability of safe and well-tolerated COVID-19 therapeutics could prevent disease progression and decrease the rate of hospitalization in vulnerable populations.

**Disclosures:**

**Rikisha Gupta, MPH**, Gilead Sciences, Inc.: Employee|Gilead Sciences, Inc.: Stocks/Bonds **Valentina Shvachko, MS**, Gilead Sciences, Inc: Employee|Gilead Sciences, Inc: Stocks/Bonds **Amos Lichtman, MPH, MD**, Gilead Sciences, Inc.: Employee|Gilead Sciences, Inc.: Stocks/Bonds **Mark Berry, PhD**, Gilead Sciences, Inc.: Employee|Gilead Sciences, Inc.: Stocks/Bonds **Florentin Scarlat, MA**, Gilead Sciences, Inc.: Employee|Gilead Sciences, Inc.: Stocks/Bonds **Anand Chokkalingam, PhD**, Gilead Sciences, Inc.: Employee|Gilead Sciences, Inc.: Stocks/Bonds

